# A Label-Free Photoelectrochemical Biosensor Based on ZnO/Cs_3_MnBr_5_ Heterogeneous Films for Alpha-Fetoprotein Detection

**DOI:** 10.3390/nano14131127

**Published:** 2024-06-29

**Authors:** Long Shao, Biyu Zhang, Wei Wu, Gengyan Cui, Mao Liu

**Affiliations:** 1 School of Metallurgy, Northeastern University, Shenyang 110819, China; 2 School of Materials Science and Engineering, Changchun University of Science and Technology, Changchun 130012, China; zhangbiyu0110@163.com (B.Z.); goodwuwei@163.com (W.W.); 3 School of Mechanical Engineering, Henan Polytechnic Institute, Nanyang 473000, China; cgy0521@126.com

**Keywords:** photoelectrochemical biosensor, ZnO/Cs_3_MnBr_5_ heterogeneous films, hepatocellular carcinoma, alpha-fetoprotein

## Abstract

Highly sensitive and specific biomarker detection is of outstanding importance for the diagnosis and treatment of cancers. Herein, we developed robust photoelectrochemical (PEC) biosensors with low background noise and high sensitivity based on a heterojunction, which can improve semiconductor photoelectric properties by limiting the recombination of photogenerated electron–hole pairs and successfully widening the range of light absorption. Alpha-fetoprotein (AFP) was used as a target model to examine the analytical performances of the designed PEC biosensors. ZnO/Cs_3_MnBr_5_ heterogeneous film with a uniform porous structure and large surface area enhanced electron transfer and biomolecule immobilization, and significantly increased the photocurrent response. Under the optimal conditions, the designed PEC biosensor exhibited a linear detection range of 0.01–500 ng/mL and a detection limit of 12 pg/mL. In addition, this PEC biosensor performed well when testing human serum samples and exhibited good repeatability, stability over time, and specificity, showing enormous potential for the detection of cancer markers in future biological and clinical research.

## 1. Introduction

Hepatocellular carcinoma (HCC) is a serious disease that poses a global threat to human life and health [1,2,3,4,5,6]. As one of the most common cancers, HCC has a 60 percent mortality rate, which is fairly significant; therefore, patients with HCC need early diagnosis and treatment [7,8,9,10]. One of the most common blood proteins in HCC is alpha-fetoprotein (AFP), a distinct tumor marker that is commonly used in HCC liver transplantation as well as HCC screening, diagnosis, treatment, and prognosis [11,12,13]. Normal human serum AFP concentrations are less than 10 ng/mL, and high AFP levels in adult serum have been associated with the development of liver cell carcinoma [9]. Hence, several techniques for detecting AFP have been developed, including electrochemical approaches [13], enzyme-linked immunosorbent assays [14,15], fluorescence immunoassays [16,17], surface plasmon resonance (SPR)-based immunoassays [18,19,20,21], and electro-chemiluminescent assays [22,23]. Despite their high sensitivity, these approaches usually require complex procedures, high costs, and lengthy processing periods. Therefore, there is a need for a novel, rapid, and highly sensitive AFP detection technology to efficiently diagnose HCC and reduce its mortality rate.

Photoelectrochemical (PEC) biosensors are widely recognized as a new and powerful technology for biological analysis, owing to their excellent affinity to biomolecules and superior inherent sensitivity compared to conventional methods [24,25]. For example, Li et al. successfully designed a novel label-free PEC biosensor based on Au/Cs_x_WO_3_ heterogeneous films for AFP detection; the as-prepared PEC biosensor had good specificity, repeatability, and long-term stability, and showed satisfactory results in human serum sample analysis [26]. This detection technique is based on the photoelectric conversion capabilities of photoactive compounds, which are used to determine the concentration of targeted biological markers [24]. In photoelectrochemical detection, appropriate light wavelengths are used to excite photosensitive substances, and electrical signals are detected. PEC detection technology is, therefore, highly sensitive and shows great promise for identifying small biomolecules and chemicals. The photocurrent, which is the output signal obtained from the photoelectric conversion of the photosensitive material in PEC biosensors, is required for the prompt detection of biomarkers. Depending on the nature of the photosensitive material, PEC biosensors can be classified into inorganic [25], organic [27,28], or composite photosensitive materials [29,30]. ZnO [31,32], TiO_2_ [33,34,35], and reduced graphene oxide [36,37] are common inorganic photosensitive materials. ZnO is a popular photocatalyst, owing to its outstanding electrical conductivity communication properties, nontoxicity, and chemical stability. However, the wide band gap of ZnO restricts its absorption to the UV region (~380 nm), limiting its application across the solar spectrum [31,38]. Furthermore, UV light has a high oxidative power that may destroy biomolecules [39]. The key challenge faced by ZnO electrodes is, therefore, the effective utilization of visible light, and coupling two materials to form a heterostructure is an effective method for enhancing the photoelectric response and overcoming this challenge [32,37,40,41].

Heterojunctions can improve semiconductor photoelectric properties by limiting the recombination of photogenerated electron–hole pairs and successfully widening the range of light absorption. To date, many studies have reported improvements in PEC activity by modifying the surface of ZnO with narrow-bandgap semiconductors, including CdX (X = S, Se, or Te) and PbS [42]. However, the heavy metal toxicity of the above-mentioned materials limits their further commercial applications. Cs_3_MnBr_5_ is a nontoxic material that has garnered research interest owing to its high visible-light absorption coefficient and optical stability for photovoltaic applications. In addition, Cs_3_MnBr_5_ is an inorganic semiconductor with outstanding chemical stability and excellent biocompatibility, which render it a good candidate for PEC biosensing of substrate [43].

**Scheme 1:**
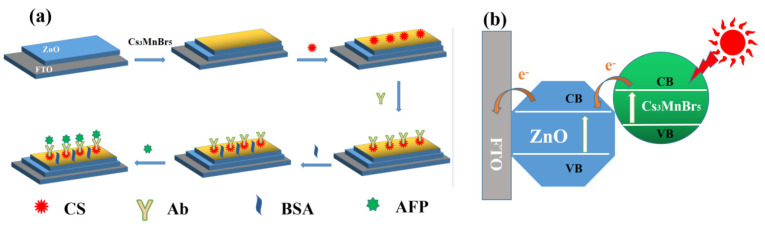
(**a**) Fabrication process of the ZnO/Cs_3_MnBr_5_ photoelectrochemical immunosensor and (**b**) its operational mechanism. VB and CB denote the valence and conduction bands, respectively.

In this study, we developed a novel ZnO/Cs_3_MnBr_5_ heterogeneous film-based label-free PEC biosensor for AFP detection (Scheme 1). The ZnO layer adopts a three-dimensional inverse opal (IO) photonic crystal structure that improves photochemical processes and solar cell photovoltaic response by increasing the effective optical path length [44]. Furthermore, the ZnO IO has a uniform porous structure and large surface area, which reduce the distance between the targeted substance and the fluorine-doped tin oxide (FTO) substrate, thereby enhancing electron transfer and biomolecule immobilization [45]. The matched effective energy level between ZnO IO and Cs_3_MnBr_5_ nanocrystals (NCs) further enables the ZnO/Cs_3_MnBr_5_ heterostructure’s full use of the visible-light spectrum, thus, facilitating charge transfer, significantly improving photocurrent responsiveness, and preventing UV damage to proteins. While the application of ZnO/Cs_3_MnBr_5_ heterostructures in PEC biosensor fabrication has not been explored to date, our experimental results indicate their immense potential for disease diagnosis.

## 2. Materials and Methods

### 2.1. Materials

Zinc nitrate (Zn (NO_3_)_2_·6H_2_O), methyl methacrylate (MMA), and glutaraldehyde (GLD) 50% were purchased from Tianjin Chemical Plant Co., Ltd. (Tianjin, China). Citric acid, ethanol, hydrogen peroxide (H_2_O_2_), and glucose were purchased from Beijing Chemical Plant Co. (Beijing, China). Tetraethoxysilane (TEOS) and sodium borohydride (NaBH_4_) were purchased from Sinopharm Chemical Reagent Co. (Beijing, China). Glucose oxidase (GOD) was purchased from Amresco Co., Ltd. (Shanghai, China). Chitosan (CS) was purchased from Solarbio Co., Ltd. (Beijing, China). Alpha-fetoprotein (AFP), carcinoembryonic antigen (CEA) prostate-specific antigen (PSA), and anti-AFP antibody (Ab, polyclonal antibody) were purchased from Beijing Boisynthese Biotechnology Co., Ltd. (Beijing, China). Bovine serum albumin (BSA, 96–99%) was purchased from Beijing DingGuo Biotechnology Company (Beijing, China). 1-ethyl-3-(3-dimethylaminopropyl) carbodiimide (EDC) was purchased from Sigma-Aldrich (Steinheim, Germany). L-cysteine was purchased from Alfa Aesar Co., Ltd. (Beijing, China). Human serum samples were purchased from a local hospital. Phosphate-buffered saline (PBS) was prepared in a conventional way to configuring the CS, Ab, BSA, and AFP to pH = 7.2 (0.01 M) and the test solution to pH = 7.4 (0.1 M), respectively. All reagents were of analytical purity and were used without further purification. All the aqueous solutions were prepared with deionized water (DI water, 18.25 MΏ/cm) that was obtained from a water purification system. Cs_2_CO_3_ (99.9%), octadecene (ODE, 90%), oleic acid (OA, 90%), oleylamine (OAm, 70%), manganese (II) acetate tetrahydrate (MnC_4_H_6_O_4_·4H_2_O, 99.999%), C_18_H_37_Br (OAmBr, 99%), toluene (ACS grade), and ODE were purchased from Macklin; others were purchased from Sigma-Aldrich, and all were used directly.

### 2.2. Apparatus

UV–visible (UV–VIS) transmittance spectra were measured using a UV-3101PC scanning spectrophotometer (Shimadzu Instruments Co., Ltd., Shanghai, China) ranging from 200 to 1100 nm. The morphology of the samples was characterized using a JSM-7500F field-emission scanning electron microscope (SEM) (JEOL Instruments Co., Ltd., Shanghai, China). Fourier transform infrared (FTIR) absorption spectra were measured using a Shimadzu DT-40 model 883 infrared spectrophotometer (Shimadzu Instruments Co., Ltd., Shanghai, China). X-ray diffractometry (XRD) analysis was conducted using a RigakuD/max 2550 X-ray diffractometer (Tiger Instruments Co., Ltd., Wuhan, China). Electrochemical impedance spectroscopies (EISs) were calculated using a model CHI660D electrochemical analyzer (ChenHua Instruments Co., Ltd., Shanghai, China). Electrochemical tests were performed with a model CHI630D electrochemical analyzer (ChenHua Instruments Co., Ltd., Shanghai, China), using a conventional three-electrode system comprising a platinum wire as a counter electrode, a saturated calomel electrode (SCE) as a reference electrode, and a modified electrode with a geometrical area of 1.0 ± 0.1 cm^2^ as a working electrode. The cyclic voltammetry curves (CVs) were recorded at a scan rate of 100 mV/s. All the photocurrent measurements were conducted under a 500 W xenon irradiation lamp at a constant potential of 0.6 V (relative to a saturated Ag/AgCl electrode) in PBS (pH = 7.4, 0.1 M).

### 2.3. Preparation of ZnO IO

Evenly monodispersed polymethyl methacrylate (PMMA) balls of a predetermined size were synthesized using a previously reported method [45]. The PMMA template was generated using a vertical-deposition self-assembly process. The obtained PMMA colloidal solution was immersed in the hydrophilic fluorine-doped tin oxide (FTO)-coated glass substrate, which was subsequently baked for 24 h at 35 °C. The surface tension generated by liquid evaporation prompted gradual self-assembly of the PMMA colloidal spheres into a highly ordered array on the FTO substrate. Subsequently, the PMMA template was sintered for 40 min at 120 °C to improve its physical properties. Figure S1 displays the morphology of the PMMA spheres.

The Zn ion precursor solution was prepared by dissolving Zn (NO_3_)_2_·6H_2_O in ethanol. Appropriate amounts of tetraethyl orthosilicate (TEOS) and citric acid were added as chelating agents, and then the solution was stirred until it became colorless and transparent. Subsequently, the precursor solution was extensively infiltrated at room temperature, and capillary forces were applied to the interstices of the PMMA templates. To obtain the ZnO IO structure, the samples were heated to 500 °C for 3 h, which burned the original PMMA template. The phase purity of the ZnO IO was determined by using X-ray powder diffraction (XRD) (Tiger Instruments Co., Ltd., Wuhan, China) (Figure S2).

### 2.4. Preparation and Modification of Cs_3_MnBr_5_ NCs

#### 2.4.1. Synthesis of Cs-Oleates

The synthesis of Cs-oleates was performed under a N_2_ atmosphere. A 25 mL three-neck round-bottom flask was filled with 0.4 g of Cs_2_CO_3_, 13 mL of octadecene (ODE), and 1 mL of oleic acid (OA). The mixture was heated at 130 °C for 1 h, or until the powder was entirely dissolved.

#### 2.4.2. Synthesis of Cs_3_MnBr_5_ NCs

The synthesis of Cs_3_MnBr_5_ NCs was performed under a N_2_ atmosphere. In a 50 mL three-neck bottle, 0.245 g MnCl_4_H_6_O_4_·4H_2_O and appropriate amounts of oleylamine, OA, oleylammonium bromide, and ODE were mixed, stirred, and the bottle filled with N_2_. The solution was heated to 140 °C for 2 h and further heated to 260 °C. Subsequently, 0.8 mL Cs-oleate was rapidly injected into the bottle and ice water was used to cool the solution, thereby producing Cs_3_MnBr_5_ NCs. Following acetone precipitation and centrifugation, the Cs_3_MnBr_5_ NCs were dissolved in toluene. Figure S3 displays the experimental flowchart. The best crystalline nanomaterials were created by optimizing the synthesis conditions (Figures S4–S6).

### 2.5. Preparation of FTO/ZnO/Cs_3_MnBr_5_ Electrode

The FTO/ZnO electrode was spin-coated with 50 μL of a 5 mL toluene solution with scattered Cs_3_MnBr_5_ NCs. The electrode was annealed at 70 °C for 1 h, dried at room temperature, and washed with phosphate-buffered solution (pH = 7.2).

### 2.6. PEC Measurements

The surface of the FTO working electrode was modified with the ZnO/Cs_3_MnBr_5_ heterogeneous layer, followed by application of 10 μL 0.05 wt% chitosan (CS) and drying at ambient temperature for 30 min. The FTO/ZnO/Cs_3_MnBr_5_ electrode was then thoroughly cleaned using NaOH solution and deionized water. After the electrode had dried for 30 min at room temperature, 20 μL of an aqueous solution containing 5% glutaric dialdehyde was added to activate the CS. Thereafter, deionized water was used to rinse the electrodes. The surface of the working electrode was subsequently altered using 25 μL of 10 μg/mL of the antibody (Ab) and kept at 4 °C for over 12 h of incubation. After removing any uncombined antibody with phosphate-buffered saline (PBS) (pH = 7.2), a 20 μL solution of 1% bovine serum albumin (BSA) was applied to the electrode surface for 30 min at 37 °C to block nonspecific active sites. After rinsing thoroughly with PBS (pH = 7.2), 25 μL solutions of the AFP antigen at various concentrations were added to the generated electrode. The electrode was incubated at 37 °C for 1 h and then thoroughly cleaned with PBS (pH = 7.2) to produce the PEC biosensor used in the subsequent experiments.

A three-electrode setup was employed for the electrochemical and PEC tests. Saturated calomel and Pt wire electrodes were used as the reference and counter electrode, respectively. The working electrode was a modified FTO electrode with an area of 1.0 ± 0.1 cm^2^. The photocurrent was measured using a constant potential of 0.6 V, supplied by a 500 W xenon lamp in a PBS solution containing 0.07 M AA (pH = 7.4) [46]. The photoelectrochemical measurement process is shown in Scheme S1.

## 3. Results and Discussion

### 3.1. Characterization

**Figure 1:**
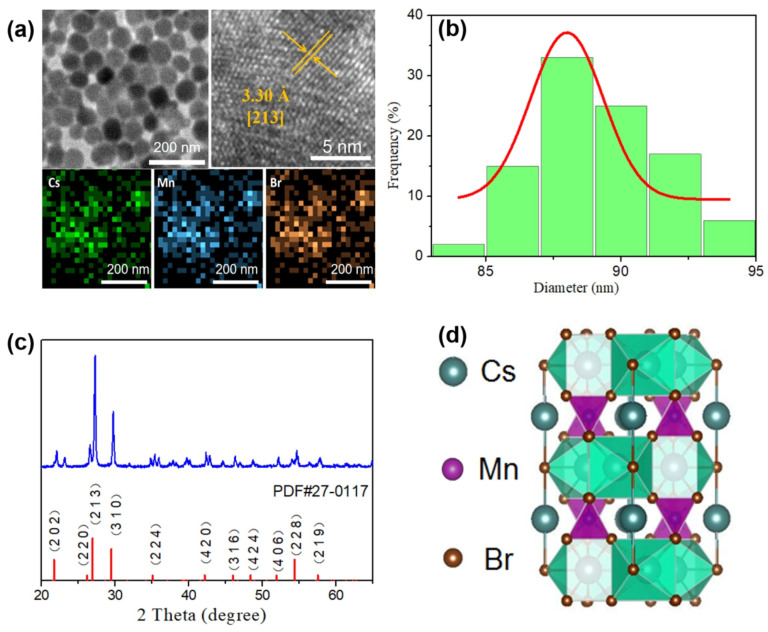
(**a**) TEM (**left**), HRTEM (**right**), and elemental mapping (**bottom**) images of the Cs_3_MnBr_5_ NCs. (**b**) Particle size statistics, (**c**) XRD pattern, and (**d**) schematic of the crystal structure of the Cs_3_MnBr_5_ NCs.

Transmission electron microscopy (TEM) was used to confirm the successful synthesis of Cs_3_MnBr_5_ NCs. The TEM image of the as-prepared Cs_3_MnBr_5_ NCs (Figure 1a) reveals that they form a regular arrangement on the TEM grid while having excellent uniformity and a particle size of approximately 87 nm. The high-resolution transmission electron microscopy (HRTEM) image reveals the lattice fringes of the [213] planes, with a distance of 3.30 Å. In addition, the elemental mapping images verify the existence of Cs, Mn, and Br in the NCs. The NC size conformed to the mathematical statistical law of Gaussian distribution, and the size was mainly distributed in the narrow range of 82–94 nm, further indicating that the NCs were monodispersed (Figure 1b). XRD measurements were utilized to determine the phase purity of the as-prepared Cs_3_MnBr_5_ NCs. TOPAS 4.2 software was used to fit the Cs_3_MnBr_5_ diffractogram line profile using the Rietveld method (Figure 1c). All peaks were indexed to a tetragonal unit cell, and their values correspond to those of the standard Cs_3_MnBr_5_ pattern (PDF#27-0117). The XRD pattern did not contain any other diffraction peaks, and the sharp signals indicate that the NCs had good crystallinity. The Rietveld refinement confirmed that the Cs_3_MnBr_5_ NCs were isostructural with Cs_3_CoCl_5_ and belonged to the tetragonal phase system with the I4/mcm space group (Figure 1d).

**Figure 2:**
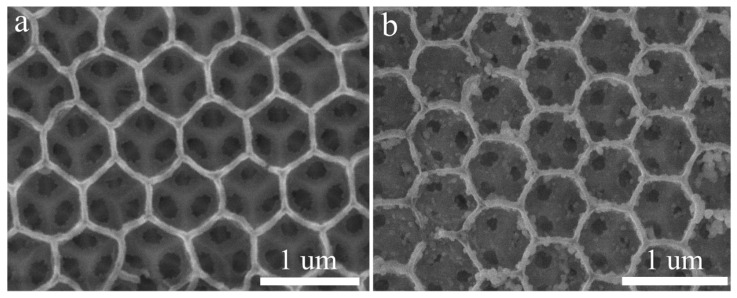
(**a**) SEM image of the FTO/ZnO Cs_3_MnBr_5_ thin films and (**b**) SEM image of FTO/ZnO/Cs_3_MnBr_5_ thin films.

The field-emission scanning electron microscopy image of the ZnO IO thin film is displayed in Figure 2a, exhibiting a pore size of approximately 700 nm and a well-organized 3D pore structure. Furthermore, a considerable number of as-prepared Cs_3_MnBr_5_ NCs adhered to the exterior and interior of the ZnO IO, as shown in the SEM image of the ZnO IO thin film modified with the Cs_3_MnBr_5_ NCs (Figure 2b). The large specific surface area of the enhanced electrode significantly enhances biological protein adsorption, hence improving the detection capacity of the sensor.

**Figure 3:**
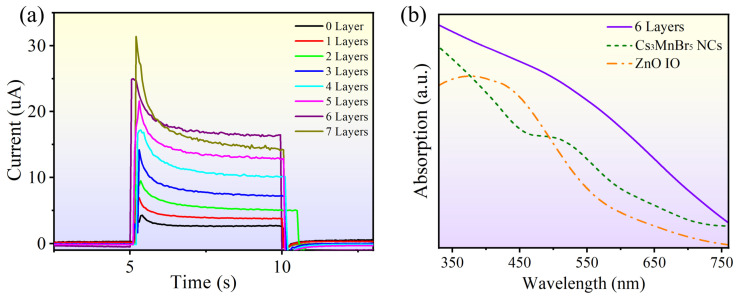
(**a**) Photocurrent of ZnO/Cs_3_MnBr_5_ composite electrodes with different number of Cs_3_MnBr_5_ NC spin-coated layers. (**b**) Absorption spectra of the ZnO IO, Cs_3_MnBr_5_ NCs, and composite electrodes coated with six layers of Cs_3_MnBr_5_ NCs.

The FTO/ZnO/Cs_3_MnBr_5_ heterogeneous film outperformed the FTO/ZnO film in terms of photocurrent responsiveness (Figure 3a). However, as the number of spin-coated Cs_3_MnBr_5_ NC layers increased to above six, the photocurrent of the composite electrode decreased. This is because the excessive deposition of Cs_3_MnBr_5_ NCs on the electrode surface increases its diffusion resistance and generates more recombination centers.

Furthermore, we investigated the spectral response capability of the ZnO/Cs_3_MnBr_5_ heterogeneous film. As shown in Figure 3b, the ZnO IO mainly absorbs in the ultraviolet range, whereas the Cs_3_MnBr_5_ NCs absorb in the visible range. Consequently, the ZnO/Cs_3_MnBr_5_ heterogeneous film absorbed in both the ultraviolet and visible-light ranges. In addition, there was more absorption in the visible-light range compared to the ZnO IO, highlighting the material’s improved photoelectric conversion efficiency.

### 3.2. Characterization of Photoelectrochemical Biosensor Construction Process

**Figure 4:**
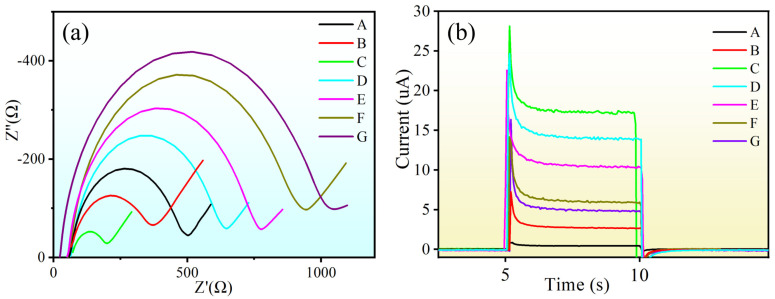
(**a**) EIS profile and (**b**) photocurrent response at each step of electrode modification: (A) FTO, (B) FTO/ZnO, (C) FTO/ZnO/Cs_3_MnBr_5_, (D) FTO/ZnO/Cs_3_MnBr_5_/CS, (E) FTO/ZnO/Cs_3_MnBr_5_/CS/Ab, (F) FTO/ZnO/Cs_3_MnBr_5_/CS/AB/BSA, and (G) FTO/ZnO/Cs_3_MnBr_5_/CS/AB/BSA/AFP.

The fabrication of the biosensor electrode was monitored using electrochemical impedance spectroscopy (EIS) and photocurrent response measurements (Figure 4). The values of electron transfer resistance (*R*_et_) were obtained by electrochemical impedance spectroscopy (EIS), which is normally used to analyze the construction process of biosensor electrodes. The *R*_et_ spectra were measured in a solution mixture that contained 0.1 M KCl and 0.05 M K_3_[Fe(CN)_6_]/K_4_[Fe(CN)_6_] (1:1) in 0.01 M PBS (pH = 7.4) buffer solution, with an open circuit voltage of 0.6 V and a frequency of 0.01–100 Hz. The *R*_et_ is equal to the diameter of the semicircle. The ZnO IO exhibited superconductivity, as shown in Figure 4a, resulting in a notable drop in electrode resistance. It was also observed that the FTO/ZnO/Cs_3_MnBr_5_ electrode showed greater *R*_et_ than the FTO/ZnO electrode. Modifying the electrode with CS caused a significant increase in the resistance because CS has an insulating property [16]. The resistance of the electrode increased slightly after further modification with Ab (25 μL, 10 μg/mL) and BSA (20 μL, 1%) because these organic molecules inhibited electron transport [45]. The impedance spectrum showed notable changes in the electrode’s surface with the use of 25 μL 10 ng/mL AFP antigen. Thus, a ZnO/Cs_3_MnBr_5_ heterogeneous film-based label-free PEC biosensor was successfully produced. The PEC biosensor was then measured using a constant potential of 0.6 V supplied by a 500 W xenon lamp in a PBS solution containing 0.07M AA (pH = 7.4).

Figure 4b shows the variations in the photocurrent response of the electrode for each processing step, and the observed photocurrent shift further validates the successful synthesis of the PEC biosensor. The results reveal that the photocurrent of the FTO/ZnO electrode increased compared to the bare FTO, confirming both the high conductivity and photoelectric conversion capabilities of the Cs_3_MnBr_5_ NCs and the successful modification of ZnO IO. The photocurrent response of the FTO substrate modified with the ZnO/Cs_3_MnBr_5_ heterogeneous film was approximately enhanced by a factor of 7, demonstrating that the heterogeneous film greatly increased the photoelectric conversion efficiency of the photoelectrochemical biosensor. The primary explanation for this enhancement is the comparable energy levels of ZnO IO and Cs_3_MnBr_5_ NCs, which enable a more efficient injection of the excited-state electrons into the ZnO IO layer, thereby facilitating charge transfer, decreasing carrier recombination, and significantly enhancing photocurrent. Furthermore, the slow-light effect of the ZnO IO can lengthen the photovoltaic response and effective optical path. These advantages have the potential to greatly improve light utilization and photoelectric conversion efficiency. The current gradually decreased after adding CS, Ab, BSA, and AFP antigens to the electrode surface due to the insulating properties of these organic compounds, which obstructed electron transfer and reduced current responsiveness [46]. The observed change in photocurrent at each stage of the modification process indicates the effective development of a label-free PEC biosensor based on the ZnO/Cs_3_MnBr_5_ heterogeneous film.

### 3.3. Optimization of the Detection Conditions

**Figure 5:**
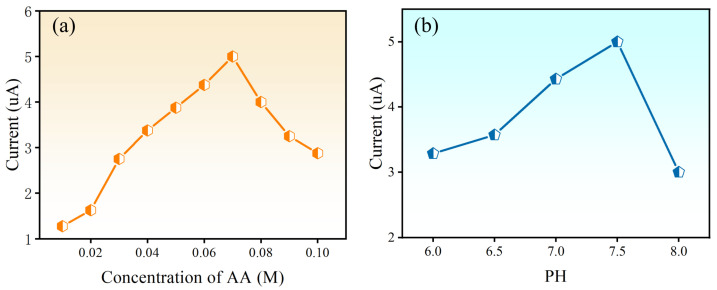
Current (**a**) with different AA concentrations and (**b**) at various pH levels (6.0–8.0). The concentrations of Ab and AFP are 10 μg/mL and 10 ng/mL, respectively.

To build a functional and optimized PEC biosensor, an experiment that involved adjusting the pH and AA levels was performed. In the detection solution, AA functioned as an electron donor to create a consistent and adequate photocurrent signal while also minimizing electron–hole recombination [47]. Therefore, the AA concentration is a crucial parameter that was studied. As shown in Figure 5a, the photocurrent intensity rises with an AA concentration of 0.02–0.07 M, whereas it decreases with an AA concentration of 0.07–0.10 M. This suggests that an AA concentration of 0.07 M provides a sufficient number of available electrons for photoinduced holes and saturated electron donors. Furthermore, the photoelectric response of the electrode is modified by the pH of the solution, which alters the biomolecular activity on the electrode [45]. A pH of 7.4 was selected as the ideal pH for identification because it produced the highest photocurrent (Figure 5b).

### 3.4. Photoelectrochemical Detection of AFP

**Figure 6:**
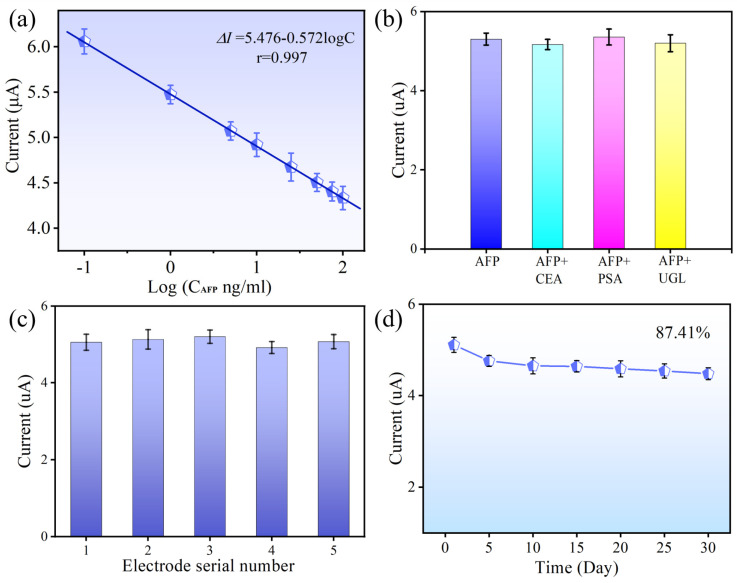
(**a**) Calibration curves of the photocurrent response of the ZnO/Cs_3_MnBr_5_ sensor for different concentrations of AFP. (**b**) Photocurrent of the sensor in the presence of different interfering substances. (**c**) Repeatability of the photocurrent response of the sensor. (**d**) Stability of the sensor over 30 days.

Under optimal conditions, variations in the AFP concentrations (25 μL) were recognized by measuring photocurrent fluctuation in the synthesized PEC biosensor. Figure 6a shows the photocurrent response calibration curve for various AFP concentrations. Notably, the photocurrent is a decreasing logarithmic function of the AFP concentration in the range of 0.01–500 ng/mL. With a determination coefficient of 0.997, the regression equation is *ΔI* = 5.476 − 0.572logC_AFP_, where *ΔI* is the photocurrent of the FTO/ZnO/Cs_3_MnBr_5_/CS/Ab/BSA heterogeneous film electrode incubated with 25 μL solutions of varying AFP concentrations. As the concentration of AFP increased, the electronic specialization of AA towards the PEC sensor was blocked, resulting in a gradual decrease in the photocurrent intensity. By calculating the signal-to-noise ratio (S/N = 3) [48], the detection limit of the PEC biosensor was found to 12 pg/mL. Our as-prepared PEC biosensor provides a larger linear range for AFP detection and a lower detection limit than heterostructure photoelectrochemical biosensors based on other materials. Table S1 shows the good performance of the PEC biosensor based on the heterogeneous ZnO/Cs_3_MnBr_5_ film.

AFP standard solutions of various concentrations were added to a standard human serum sample to determine the relative standard deviation (RSD). Table S2 shows that the analytical recovery range is 95–104%, with RSD values smaller than 5.3%. These findings demonstrate that the ZnO/Cs_3_MnBr_5_ heterogeneous film-based label-free PEC biosensor is very sensitive and accurate for AFP detection in real samples.

### 3.5. Specificity, Reproducibility, and Stability of the PEC Biosensor

The selectivity of the proposed AFP biosensor was evaluated by adding other interfering species, including carcinoembryonic antigen, prostate-specific antigen, and uteroglobin. The three electrodes treated with solutions containing 100 ng/mL interfering protein and 10 ng/mL AFP produced photocurrent responses due to the specific binding (Figure 6b). The photocurrent did not vary significantly, indicating that nonspecific adsorption had no effect on the PEC biosensor. As a result, the ZnO/Cs_3_MnBr_5_ heterogeneous film-based label-free PEC biosensor exhibited excellent specificity.

Five PEC biosensors were created under identical experimental conditions to assess their photoelectric response at 10 ng/mL of AFP. As shown in Figure 6c, there was little variance in the photocurrent intensity generated by the five PEC biosensors, demonstrating the reproducibility of the ZnO/Cs_3_MnBr_5_ heterogeneous film-based label-free PEC biosensor’s performance. The long-term stability of a ZnO/Cs_3_MnBr_5_ heterogeneous film electrode that was kept in a refrigerator at 4 °C is displayed in Figure 6d. Every five days, the photoelectric response of this sensor was tested, using 10 ng/mL of AFP. After 30 d, the electrode retained 87.41% of its initial performance, and the photocurrent slightly decreased with storage time, demonstrating the strong long-term durability of the ZnO/Cs_3_MnBr_5_ heterogeneous film-based label-free PEC biosensor.

## 4. Conclusions

In this study, a ZnO/Cs_3_MnBr_5_ heterogeneous film was synthesized to effectively create a novel label-free PEC biosensor for AFP detection. The delayed light effect in the ZnO IO improved the photovoltaic performance of the electrode in solar cells and photochemical processes by increasing the effective optical path. The ZnO/Cs_3_MnBr_5_ heterogeneous film leverages the homogeneous porosity structures of the ZnO IO due to its large surface area for the immobilization of biomolecules and electron transport. The addition of Cs_3_MnBr_5_ NCs increases the photoelectric response of the ZnO IO, allowing it to fully utilize visible light, stimulating charge transfer, and significantly increasing the photocurrent response. We also demonstrated the use of a ZnO/Cs_3_MnBr_5_ heterogeneous film in a photoelectrochemical bioassay for the first time. Under ideal conditions, a linear detection range of 0.01–500 ng/mL with a detection limit of 12 pg/mL was noted. Additionally, the as-prepared PEC biosensor performed well while testing human serum samples and exhibited good repeatability, stability over time, and specificity. Therefore, our ZnO/Cs_3_MnBr_5_ heterogeneous film-based label-free photoelectrochemical biosensor has enormous potential for the identification of cancer markers in biological and clinical research.

## Data Availability

The data presented in this study are available on request from the corresponding author.

## Supplementary Materials

The following supporting information can be downloaded at: https://www.mdpi.com/article/10.3390/nano14131127/s1. Figure S1: SEM image of PMMA spheres; Figure S2: XRD image of ZnO IO; Figure S3: Schematic diagram of Cs_3_MnBr_5_ NCs synthesis; Figure S4: (a) TEM images, (b) XRD, and (c) Luminescence of synthesized products at different reaction temperatures; Figure S5: (a) TEM images and (b) Luminescence spectrum of the synthesized product with varying amounts of OA; Figure S6: (a) TEM images and (b) Luminescence spectrum of the synthesized product with varying amounts of OAm; Scheme S1: The photoelectrochemical measurement process; Table S1: Performance comparison of heterostructure photoelectrochemical biosensors based on other materials for AFP detection; and Table S2: Detection of AFP in human standard serum. Reference [49] are cited in the Supplementary Materials.
