# Supplementary material for: A Label-Free Photoelectrochemical Biosensor Based on ZnO/Cs3MnBr5 Heterogeneous Films for Alpha-Fetoprotein Detection
Source: Nanomaterials (Basel). 2024 Jun 29;14(13):1127. doi: 10.3390/nano14131127 (PMC11243747; doi:10.3390/nano14131127)
Supplement: Supplementary file 1 [file nanomaterials-14-01127-s001.zip › nanomaterials-3040923-supplementary.pdf]

nanomaterials

Supplementary Materials

A Label-Free Photoelectrochemical Biosensor Based on ZnO/Cs_3MnBr_5 Heterogeneous Films for Alpha-Fetoprotein Detection

Long Shao [1,2,*], Biyu Zhang [2], Wei Wu [2], Gengyan Cui [3] and Mao Liu [1,*]

[1] School of Metallurgy, Northeastern University, Shenyang 110819, China
[2] School of Materials Science and Engineering, Changchun University of Science and Technology, Changchun 130012, China
[3] School of Mechanical Engineering, Henan Polytechnic Institute, Nanyang 473000, China
* Correspondence: shaolong@smm.neu.edu.cn (L.S.); liumao@mail.neu.edu.cn (M.L.)

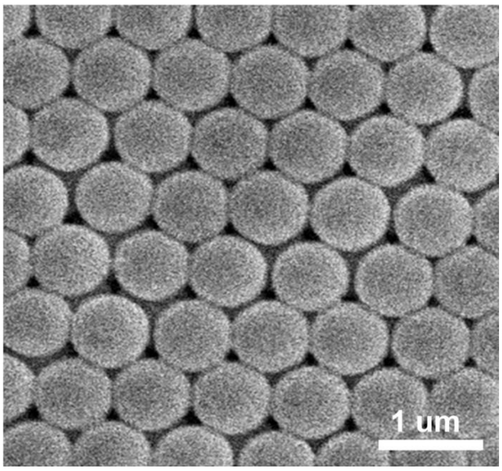

Figure S1: SEM image of PMMA spheres.

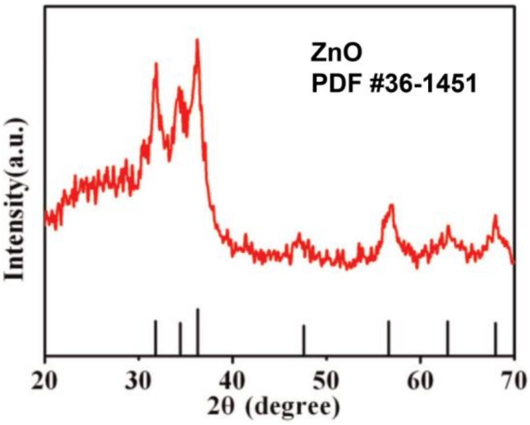

Figure S2: XRD image of ZnO IO.

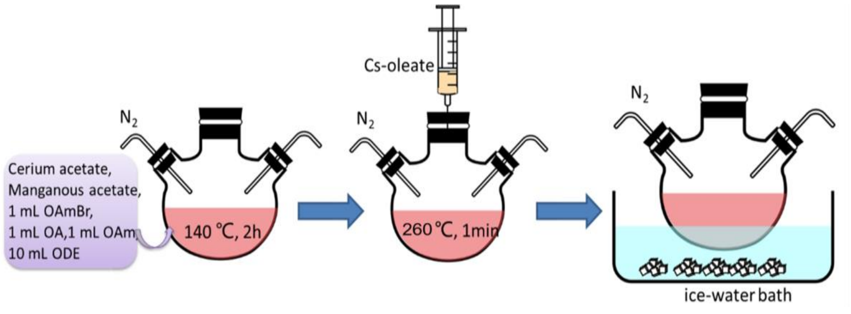

Figure S3: Schematic diagram of Cs_3MnBr_5 NCs synthesis

Synthesis and regulation of Cs_3MnBr_5 NCs.

During the preparation of Cs_3MnBr_5 NCs, we achieved the regulation of the morphology and luminescence properties by adjusting the reaction temperature and the amount of ligands (oleic acid and oleylamine).

First, when the amounts of oleic acid and oleylamine remain constant (OA is 1mL and OAm is 1mL), the growth of crystals is adjusted by changing the reaction temperature. As shown in Figure S3, the synthesis temperature during the reaction process gradually increases from 160 °C to 300 °C. Through TEM and XRD analysis, it can be seen that when the reaction temperature is in the range of 160-200 °C, the synthesized product has a flower-like crystal structure. Through XRD analysis, it can be seen that the synthesized product is cesium bromide (CsBr) crystal, and Cs_3MnBr_5 NCs cannot be formed. This is because at low temperatures, the Mn-OAc bond is not easily broken to achieve cleavage, so the [CsBr8] polyhedral structure cannot be formed, and ultimately Cs_3MnBr_5 NCs cannot be formed. When the reaction temperature is in the range of 200-220 °C, it can be seen through XRD analysis that Cs_3MnBr_5 NCs gradually form in the experiment, but the crystal phase is not pure and there is still a large amount of CsBr. When the temperature reaches 240 °C, the Mn-OAc bond is completely destroyed and Cs-Br bonds are formed, forming a [CsBr8] polyhedral structure. Finally, we obtain pure Cs_3MnBr_5 NCs. When the temperature exceeds 280 °C, it can be seen from the fluorescence emission spectrum that the luminescence of the nanoparticles slightly weakens. At the same time, through XRD analysis, it can be seen that no new substances are formed in the experiment, indicating that the increase in temperature only deteriorates the crystallinity of the final product. Here we believe that it may be due to excessive overflow of ligand (OA, OAm) vapor during high-temperature heating, which increases the surface defects of the formed nanoparticles and affects the crystallinity of the experimental sample, resulting in a decrease in luminescence of the nanomaterials.

Then, we studied the luminescence properties of Cs_3MnBr_5 NCs under 365 nm ultraviolet excitation. The spectrum shows that there is almost no fluorescence emission at 160-180 °C, further indicating that no Cs_3MnBr_5 NCs are formed. During the process of 200-260 °C, the luminescence phenomenon gradually increases from weak to maximum, indicating that Cs_3MnBr_5 NCs are gradually formed. When the temperature exceeds 280 °C, the luminescence of nanoparticles slightly weakens through fluorescence emission spectroscopy. Meanwhile, through XRD analysis, it is found that no new substances are formed in the experiment, indicating that the increase in temperature only deteriorates the crystallinity of the final product. Here we believe that it may be due to excessive nitrogen gas entering during high-temperature heating, which causes excessive overflow of ligand (OA, OAm) vapor. The reduction of ligands increases the surface defects of the formed nanoparticles, which in turn affects the crystallinity of the experimental sample,

resulting in weakened luminescence of the nanomaterial and ultimately affecting the optical properties of the material.

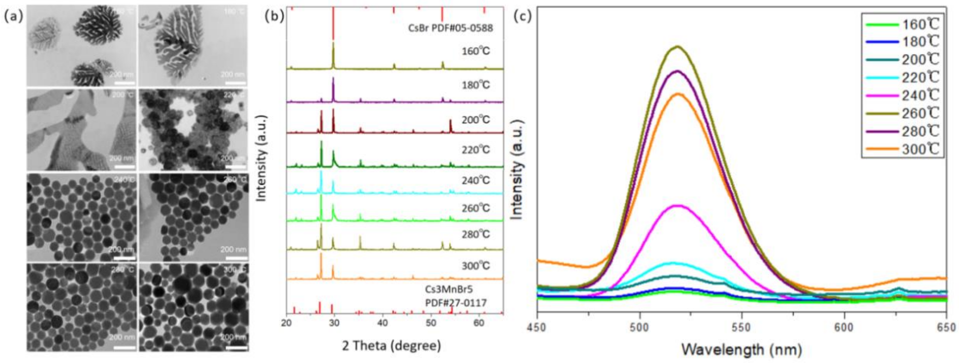

Figure S4: (a) TEM images, (b) XRD, (c) Luminescence of synthesized products at different reaction temperatures.

Further discuss the influence of the amount of ligands (OA, OAm) on the luminescence of Cs_3MnBr_5 NCs during the reaction process when the reaction temperature remains constant at 260 °C. As shown in Figure S4, when the amount of OA remains constant at 1mL and the amount of OAm is less than 1ml, the luminescence is weak; as the amount of OAm increases, the luminescence gradually increases; when the amount of OAm is excessive, the luminescence remains basically unchanged. Subsequently, we conducted in-depth analysis of the experimental phenomena: when the amount of OAm ligands is too low, this experimental environment is not conducive to the formation of crystals, resulting in excessive defects in Cs_3MnBr_5 NCs, which greatly affects the optical properties of the material; when the amount of OAm is excessive, the excessive amount of OAm ligands does not affect the formation of crystals, and OAm can also effectively reduce the surface defects of crystals and improve the stability of nanomaterials.

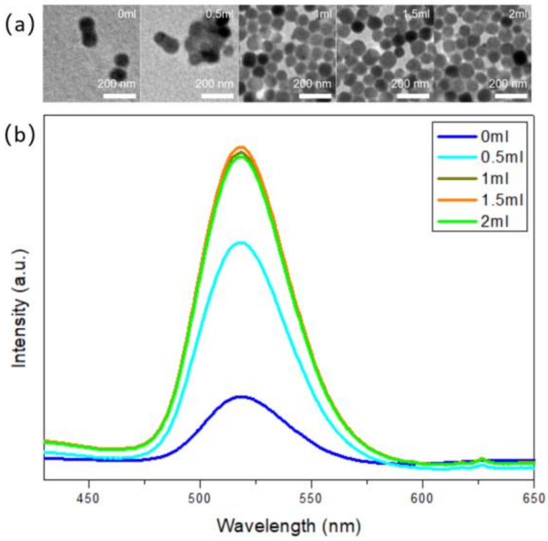

Figure S5: (a) TEM images and (b) Luminescence spectrum of the synthesized product with varying amounts of OAm.

Subsequently, the influence of the amount of OA on the properties of the synthesized product during the reaction process was analyzed. During the reaction process, when

OAm remained constant at 1 mL and the reaction temperature was 260 °C, it can be seen from Figure S5 that when the amount of OA is less than 1 mL, the luminescence is very weak; as the amount of OA increases, the luminescence gradually increases; when the amount of OA is 1 mL, the luminescence intensity reaches its maximum value; however, when the amount of OA is excessive, the luminescence significantly weakens. Further analysis shows that when OA is used as a surfactant, it not only has a reducing property but also provides a stable environment for chemical synthesis, so the lack of oleic acid in the reaction is not conducive to the formation of crystals. However, compared with OAm, OA has a stronger binding effect, so when it is excessive, it significantly affects the crystallinity of the product.

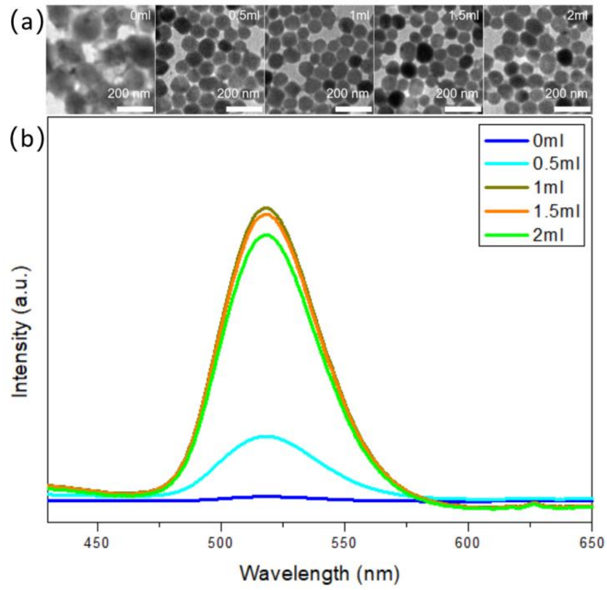

Figure S6: (a) TEM images and (b) Luminescence spectrum of the synthesized product with varying amounts of OA.

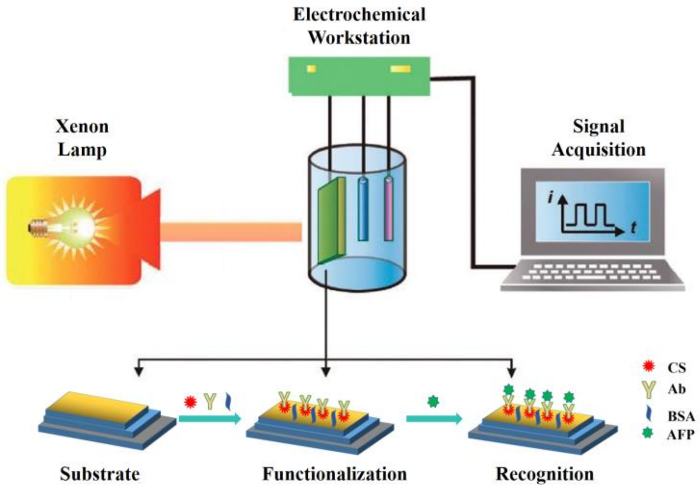

Scheme S1: The photoelectrochemical measurement process.

Table S1: Performance comparison of heterostructure photoelectrochemical biosensors based on other materials for AFP detection.

Electrode	Detection (ng/mL)	LOD (pg/mL)	Ref.
ZnO/CdS	0.05-200	8	[46]
ZnO/Ag2S	0.05-100	40	[45]
ZnO/Ag/NaYF4: Yb	51.25	5.03	[49]
Au/Cs_xWO_3	102	4.64	[26]
ZnO IOs/Cs_3MnBr_5	105.10	4.75	This work

Table S2: Detection of AFP in human standard serum.

Added (ng/mL)	Measured (ng/mL)	RSD (%, n=5)	Recovery (%)
0	1.31	5.22	-
1	2.32	4.36	95.7
10	12.02	4.96	99.3
50	52.31	5.12	103.7
100	103.06	4.71	101.6

References

49 Chen, X.; Xu, W.; Jiang, Y.; Pan, G.; Zhou, D.; Zhu, J.; Wang, H.; Chen, C.; Li, D.; Song, H. A novel upconversion luminescence derived photoelectrochemical immunoassay: ultrasensitive detection to alpha-fetoprotein. *Nanoscale* **2017**, *9*, 16357-16364, doi:10.1039/c7nr05577c.
